# CRT-D or CRT-P: When There Is a Dilemma and How to Solve It

**DOI:** 10.3390/jcm14196933

**Published:** 2025-09-30

**Authors:** Ageliki Laina, Christos-Konstantinos Antoniou, Dimitrios Tsiachris, Athanasios Kordalis, Petros Arsenos, Ioannis Doundoulakis, Polychronis Dilaveris, Anastasia Xintarakou, Panagiotis Xydis, Stergios Soulaidopoulos, Aikaterini-Eleftheria Karanikola, Nikias Milaras, Skevos Sideris, Stefanos Archontakis, Apostolos Vouliotis, Ourania Kariki, Constantinos Tsioufis, Konstantinos Gatzoulis

**Affiliations:** 1 First Department of Cardiology, “Hippokration” Hospital, National and Kapodistrian University of Athens, 11527 Athens, Greece; agelikilaina@hotmail.com (A.L.); dtsiachris@yahoo.com (D.T.); akordalis@gmail.com (A.K.); anast.xint@gmail.com (A.X.); apostolosvouliotis@gmail.com (A.V.);; 2 Third Department of Cardiology, Sotiria Thoracic Diseases General Hospital, National and Kapodistrian University of Athens, 11527 Athens, Greece; 3 State Department of Cardiology, “Hippokration” General Hospital of Athens, 11527 Athens, Greece; 4 Department of Cardiology, Onassis Cardiac Surgery Center Athens, 17674 Athens, Greece

**Keywords:** CRT-D, CRT-P, sudden cardiac death

## Abstract

Cardiac resynchronization therapy (CRT) represents a cornerstone in the management of patients with heart failure and electrical dyssynchrony, improving symptoms, reducing hospitalizations, and prolonging survival. CRT can be delivered via a pacemaker (CRT-P) or an ICD (CRT-D). Despite its widespread use, the mortality benefit of CRT-D over CRT-P remains uncertain, as no head-to-head randomized trials have been designed to directly compare the two modalities, making device selection a frequent clinical dilemma. In practice, CRT-D accounts for 70–80% of CRT implantations in developed countries, yet solid evidence demonstrating its superiority over CRT-P is lacking. Specific patient groups, including those with non-ischemic cardiomyopathy, advanced age, multiple comorbidities, or limited life expectancy, may derive limited incremental benefit from CRT-D, which should be balanced against device costs and specific risks such as lead failure and inappropriate shocks. The present review aims to provide a comprehensive comparison between CRT-D and CRT-P, focusing on the existing body of evidence, criteria for patient selection, comparative clinical outcomes, and risk–benefit considerations for clinical decision-making.

## 1. Introduction

Heart failure remains a leading cause of morbidity and mortality worldwide, affecting 1–2% of the adult population, with a rising prevalence mainly due to ageing [1,2,3]. Patients with heart failure and left ventricular dysfunction commonly exhibit significant intraventricular conduction delay, resulting in electrical dyssynchrony, exacerbating cardiac function [4,5]. It has been reported that intraventricular conduction delays (IVCDs) occur in 15% to 30% of patients with chronic heart failure with reduced ejection fraction (HFrEF) [6,7]. Cardiac Resynchronization Therapy (CRT) has emerged as a cornerstone therapeutical intervention for such patients, aiming to achieve coordinated biventricular contraction and improve hemodynamic performance [8,9,10,11], raising the number of CRT implantations up to 76% from 2010 to 2019 [12]. Multiple randomized clinical trials (RCTs) have shown reduced mortality and hospitalizations as well as improvement of symptoms and quality of life in patients with heart failure and reduced left ventricular ejection fraction (LVEF) receiving a CRT device [8,9,11,13,14].

CRT can be delivered via cardiac resynchronization therapy with a pacemaker (CRT-P) and cardiac resynchronization therapy with a defibrillator (CRT-D). The mortality benefit of CRT-D compared to CRT-P remains uncertain, primarily due to the absence of head-to-head RCTs specifically designed to compare these two therapeutic modalities. Selecting the most appropriate device type requires an individualized risk assessment that takes into account multiple factors, including the underlying etiology, presence of myocardial scar tissue, patient age, comorbidities, and overall life expectancy. ICD-specific risks such as inappropriate shocks, lead failure, and high cost should also be considered.

This review aims to provide a comprehensive comparison between CRT-D and CRT-P, focusing on the existing body of evidence, criteria for patient selection, comparative clinical outcomes, and key considerations for clinical decision-making.

## 2. Clinical Effectiveness of CRT

Current understanding and clinical use of CRT is based on landmark RCTs conducted since 2002. The MIRACLE trial was the first prospective study to demonstrate the effectiveness of CRT over a six-month follow-up period in patients with moderate-to-severe heart failure and intraventricular conduction delay compared with medical treatment [13]. Similar results were reported in the CARE-HF trial, where patients at NYHA III-IV receiving CRT experienced significant improvement in quality of life and clinical outcomes as opposed to those treated with medication alone [15,16]. Along this line, patients with advanced heart failure, wide QRS interval, and CRT exhibited reduced combined risk of death from any cause or hospitalization in the COMPANION trial, the only study to randomize patients to CRT-D or CRT-P; however, the study was designed to assess the effectiveness of CRT compared to medical treatment [9]. Patients with mild heart failure (NYHA I-II) also benefit from CRT, as shown in the MADIT-CRT [10], RAFT [11], and REVERSE [14] clinical trials. The major clinical trials of CRT are summarized in Table 1.

## 3. CRT Indications

CRT is recommended in selected patients with heart failure who remain symptomatic despite optimal guideline-directed medical therapy (OGMT). According to the 2021 ESC Guidelines on cardiac pacing and resynchronization therapy, CRT-D is indicated in patients with left ventricular ejection fraction (LVEF) ≤ 35%, sinus rhythm, and a widened QRS complex, particularly with left bundle branch block (LBBB) morphology [23]. The greatest benefit is observed among those with QRS duration ≥ 150 ms. Additionally, patients with atrial fibrillation (AF) may be candidates for CRT, given that adequate biventricular pacing can be ensured, either with medical treatment or following AV nodal ablation [23]. Patients who have received a conventional ICD and who subsequently develop symptomatic HF with LVEF ≤ 35% despite OGMT, and who have a significant proportion of RV pacing, should be considered for CRT upgrade [24,25]. In Table 2, we summarize available current guidelines regarding cardiac physiologic pacing indications.

## 4. CRT-D or CRT-P

Should all patients with a CRT indication receive a defibrillator lead? The choice between the two is a frequent clinical dilemma. Recent survey studies have shown that CRT-D devices account for over 70–80% of all CRT implantations in developed countries [26,27,28]. However, this widespread use of CRT-D is not supported by solid evidence demonstrating its superiority over CRT-P.

In a post hoc analysis of the COMPANION study, CRT-D was associated with 36% mortality risk reduction compared to CRT-P, and regarding the cause of death, CRT-D significantly reduced sudden cardiac death (SCD) (HR 0.44, 95% CI 0.23–0.86; *p* = 0.02) compared to CRT-P (HR 1.21, 95% CI 0.7–2.07; *p* = 0.50) [9]. Importantly, though, the COMPANION trial was underpowered to detect a survival benefit from CRT-D. A network meta-analysis of 13 randomized clinical trials including >12,000 patients found that CRT-D reduced total mortality by 19% (95% CI 1–33%, unadjusted) compared with CRT-P [29]. Similar results were reported in a propensity-matched cohort, where CRT-D was associated with significantly lower all-cause mortality than CRT-P in patients with heart failure of ischemic etiology and in patients with non-ischemic heart failure below 75 years of age [30]. Accordingly, recently published meta-analysis of 26 observational studies, including 55,469 CRT-P patients and 72,561 CRT-D patients, showed that patients with CRT-D had 26% lower risk of all-cause mortality compared with CRT-P. However, patients aged > 75 years old and those with non-ischemic heart failure were less likely to benefit from a CRT-D [31]. Along this line, a previous meta-analysis focused on non-ischemic cardiomyopathy (NICM) reported that the addition of a defibrillator was not significantly associated with a reduction in all-cause mortality in CRT-eligible patients [32]. The DANISH trial had similarly reported no significant difference in mortality risk in patients with NICM between the ICD and no-ICD arm, irrespective of CRT [33]. Likewise, data from observational studies from Kutyifa et al. and Barra et al. suggest substantial (24–30%) mortality benefit from CRT-D only in patients with ischemic cardiomyopathy [34,35]. C-However, contradictory results were reported in another meta-analysis assessing the effect of CRT-P versus CRT-D on mortality in patients with NICM, where CRT-D was associated with significantly lower all-cause mortality (log HR − 0.169, SE 0.055; *p* = 0.002) compared to CRT-P [36]. CRT with pacing only was reported to be non-inferior to CRT-D in the retrospective observational RESET-CRT study in an overall population of 3569 patients with both ischemic and non-ischemic heart failure [22].

Although CRT-D may offer additional survival benefit over CRT-P, reducing sudden cardiac death (SCD) risk [9], there is data that CRT-P alone could confer SCD risk reduction. In particular, in the CARE HF extended study CRT-P reduced SCD risk by 5.6% [37]. Accumulating data from subgroup analyses from RCTs suggest that SCD risk is related to the extent of reverse LV remodeling with CRT; thus, CRT responders are at lower risk for malignant ventricular arrhythmias and SCD than non-responders [38,39]. Super responders have been described as more likely having nonischemic etiology with complete left bundle branch block, and possibly women, and are characterized by significant LVEF improvement to nearly normal [40]. A downgrade to CRT-P at the time of generator replacement has been proposed, given the favorable prognosis of the super responders and low risk of ventricular arrhythmia [41]. However, appropriate ICD shocks have been described in super responders, indicating that LVEF improvement alone is not sufficient to accurately stratify SCD risk [42,43]. Women are shown to be better responders, with a higher percentage of biventricular pacing, decreased rates of death, and fewer hospitalizations compared to male counterparts, although they are underrepresented in CRT trials and are less likely to receive CRT-D [44]. Current medical treatment may mitigate ventricular arrhythmia risk, particularly the use of sacubitril/valsartan and sodium–glucose co-transporter-2 inhibitors (SGLT2i) [45,46]. Increasing comorbidities, on the other hand, are associated with a mortality risk that competes with sudden arrhythmic death. The incremental benefit of an ICD is questioned in certain clinical settings. High comorbidity burden such as advanced age, chronic kidney disease, diabetes, and peripheral vascular disease is significant predictor of mortality in CRT-D recipients, attenuating the survival benefit of ICD therapy [47,48,49,50]. Data from observational studies suggest that the addition of ICD has no impact on survival in elderly patients undergoing implantation of a CRT device [51,52]. Adding ICD to CRT seems to be a better option for younger patients with good survival prognosis. Contrast-enhanced CMR-guided scar adds valuable information concerning the risk of ventricular arrhythmia. According to the Gaudi CRT study, the presence of myocardial scar independently can predict appropriate ICD therapies and SCD in CRT patients [53]. Similarly, patients with NICM and left ventricular midwall fibrosis in CMR benefit from more from CRT-D than CRT-P [54].

As no head-to-head RCTs directly comparing CRT-D to CRT-P have been developed, the mortality benefit of CRT-D is not established. While both patients with CRT-P and those with CRT-D share common risks such as lead dislodgement, pocket hematoma, venous complications, device malfunction, and infection, CRT-D carries a higher overall complication burden [55]. This is mainly due to increased rates of late lead- and generator-related problems and increased occurrence of infections. Inappropriate shocks and psychological distress should be taken into account, along with significantly higher costs involved due to greater complexity, hardware requirements, and shorter device runtime, needing more frequently battery replacement [23].

It is therefore crucial to reassess and refine patient selection criteria in order to identify which individuals will truly benefit from the addition of a defibrillator component. Table 3 and Table 4 summarize existing studies and meta-analyses, respectively, on the effect of CRT on mortality in patients with ischemic and non-ischemic cardiomyopathy. 

## 5. Our Two-Step Approach

A two-step, multifactorial, electrophysiology (EP)-guided approach was proposed for risk stratification and management of post-myocardial infarction patients [42]. According to current guidelines, ICD is suggested in patients with LVEF < 35% for primary prevention. However, the PRESERVE EF study revealed a high-risk subpopulation among those with preserved LVEF, who received an ICD following inducible sustained monomorphic ventricular tachycardia during PVS. The authors proposed a stepwise approach involving the assessment of non-invasive risk factors (NIRFs), specifically (i) >30 premature ventricular complexes/hour on 24 h electrocardiography, (ii) presence of non-sustained ventricular tachycardia on 24 h electrocardiography, (iii) 2/3 positive criteria for late potentials, either conventional or modified, (iv) QTc derived from 24 h electrocardiography >440 ms (men) or >450 ms (women) according to the Fridericia formula from a signal recorded in three pseudo-orthogonal leads, (v) ambulatory T wave alternans ≥65 μV in two Holter channels, (vi) standard deviation of normal RR intervals ≤75 ms on the 24 h electrocardiography, and (7) deceleration capacity ≤4.5 ms, heart rate turbulence onset ≥0%, and heart rate turbulence slope ≤2.5 ms [42,77]. The presence of at least one NIRF leads to subsequent programmed ventricular stimulation (PVS), and according to inducibility of sustained monomorphic ventricular tachycardia (SMVT), to ICD implantation. In the PRESERVE-EF study by Gatzoulis et al., late potentials and the presence of non-sustained ventricular tachycardia had a higher predictive value for inducibility during PVS among the studied NIRFs. On the contrary, the recent REFINE-ICD trial randomized post-myocardial infarction patients with LVEF 36–50% and abnormal ECG markers (impaired heart rate turbulence and abnormal T wave alternans) to an ICD plus medical therapy or medical therapy alone. While the presence of abnormal ECG markers was associated with a higher rate of death, survival was not improved with ICDs compared with medical therapy alone [42]. Risk stratification for SCD in NICM is also traditionally based on LVEF, although LVEF as a sole criterion cannot accurately identify truly high-risk individuals [78]. The results from the DANISH trial questioned the utility of ICDs in NICM and low LVEF [33], while at the same time, considerable SCD risk exists among those with mildly reduced and preserved LVEF [79,80]. A similar approach to the ICM two-step EP-guided risk stratification approach has been suggested for patients with NICM and preserved ejection fraction, with pending results [81,82]. Unexplained syncope and LGE presence have been added to the risk stratification algorithm in the ReCONSIDER study [81,83]. A recently published comparative analysis of ICD efficacy in patients with ICM and NICM showed clear benefit in ICM, whereas no significant reduction in mortality or ventricular arrhythmias was shown in NICM [84]. Thus, patients with CRT-D indication who may not clearly benefit from a defibrillator lead, such as the elderly, patients with multiple comorbidities, or patients with NICM, could be subjected to this multifactorial strategy in order to assess SCD risk, facilitating decision making. Likewise, patients with a pacemaker who become eligible for CRT upgrade could be subjected to non-invasive programmed stimulation (NIPS) via the device in order to assess arrhythmic risk and proceed to CRT-D implantation [85].

Refinement of patient selection criteria is particularly warranted in NICM, where adjunctive tools such as CMR and genetic testing are gaining ground and together with EPS provide valuable guidance for risk stratification and clinical decision-making [86,87]. CMR provides reliable information about biventricular function and myocardial substrate through late gadolinium enhancement (LGE) and advanced tissue mapping techniques. The extent and distribution of myocardial scar, especially in ischemic cardiomyopathy, are closely linked to the risk of ventricular arrhythmias and sudden cardiac death; in such patients, the addition of defibrillator therapy may be justified. In NICM, mid-wall fibrosis detected by LGE or diffuse fibrosis identified by T1 mapping has also been associated with increased arrhythmic risk, potentially favoring CRT-D over CRT-P. A range of CMR parameters have been associated with SCD, including the presence and extent of LGE, T1 relaxation times, and myocardial strain [88,89]. For example, 252 patients with NICM and CRT, of whom 68 had LGE, were prospectively followed, and it was observed that CRT-D was associated with significantly higher survival than CRT-P only in patients with LGE. In patients without LGE, with their low arrhythmic risk, CRT-D offered no benefit compared with CRT-P [12]. Parallel advances in cardiogenetics have improved our understanding of the complex genetic architecture of dilated cardiomyopathy [90]. Identifying a causative gene variant in a patient with DCM improves prognostic accuracy regarding disease progression and may contribute to the indications for device implantation. Specifically, variants in genes such as *LMNA*, *RBM20*, *PLN*, and *BAG3* are consistently associated with a worse prognosis and are recognized as risk modifiers for the primary prevention of sudden cardiac death [91,92]. Similarly, pathogenic or likely pathogenic variants in *FLNC* or desmosomal genes (e.g., *DSP*, *DES*) confer an increased susceptibility to ventricular arrhythmias and/or progression to heart failure [93]. Of note, previously published guidelines for the management of patients with ventricular arrhythmias and the prevention of sudden cardiac death proposed PVS as a risk factor along with syncope, LGE in CMR, and pathogenic mutations in certain genes in the suggested SCD risk stratification algorithm [94]. Currently, artificial intelligence (AI) is emerging as a complementary tool to imaging and genomics in cardiomyopathies [90,95]. By integrating multimodal data, AI may provide new insights, advancing our understanding of cardiomyopathies and potentially refining the choice between CRT-D and CRT-P. Although early in clinical use, AI offers a promising path toward precision medicine.

## 6. CRT Non-Responders

Non-responders to CRT comprise a rather non negligeable amount of CRT receivers, and nonresponse to CRT has been related to right ventricular dysfunction. Approximately 30% of patients fail to exhibit clinical or echocardiographic improvement with CRT [96], partly attributed to the non-physiologic electrical resynchronization between an epicardial wavefront from the CS lead and the RV endocardium, suboptimal lead position, presence of LV scar, and latency due to localized conduction delay [97]. Moreover, in 5–7% of cases, CS lead implantation may be unsuccessful because of anatomic challenges, high pacing thresholds, or phrenic nerve stimulation [98]. Several approaches have been proposed to address this issue, including direct pacing of the conduction system (His bundle or left bundle branch pacing), pacing the left ventricle from multiple sites within the coronary sinus (multipoint pacing), or preferential LV pacing [99,100,101].

It is noteworthy that biventricular pacing may be complicated by ventricular proarrhythmia in the early post-implantation period; a rare but clinically significant phenomenon [102,103,104,105,106]. For example, VT storm occurred in 4% of the 191 patients included in a prospective study examining the incidence of VT storm after CRT-D implantation [104]. Similarly, 5 of 145 consecutive patients (3.4%) receiving a CRT device over a 4-year period experienced ventricular tachyarrhythmia after initiation of biventricular pacing in a case series study [107]. The causes of the proarrhythmogenicity are multiple. The reversal of the direction of the activation of the LV wall, from the epicardium to the endocardium, results in prolongation of repolarization that may trigger polymorphic VT. Secondarily, pacing close to or within myocardial scar and regions of slow conduction may increase the likelihood of VT. CRT responders, though, are less likely to experience ventricular proarrhythmia compared to non-responders [108]. Data suggest a modest effect of biventricular pacing on the incidence of new-onset atrial fibrillation as well [102]. Conduction system pacing through more physiological pacing and more effective resynchronization promotes remodeling, providing a less arrhythmogenic substrate compared with biventricular pacing.

## 7. Role of Conduction System Pacing

Conduction system pacing (CSP)—whether His bundle pacing (HBP) or left bundle branch area pacing (LBBAP)—has emerged as a possible alternative to achieve cardiac resynchronization [99,109,110,111]. HBP has been associated with higher pacing thresholds, lower implantation success rates, and significant rates of crossover to other pacing modalities [112,113]. Recently, LBBAP has been proposed as viable and effective alternative to HBP, offering higher procedural success and lower pacing thresholds [114,115]. In LEVEL-AT, a single-center, prospective, randomized, parallel, controlled, clinical trial patients allocated to biventricular pacing or CSP (either HBP or LBBP) presented similar degrees of cardiac resynchronization, ventricular reverse remodeling, and clinical outcomes [99]. LBBP-CRT demonstrated greater LVEF improvement than BiVP-CRT in heart failure patients with nonischemic cardiomyopathy and LBBB in a prospective randomized trial of 40 patients [109]. Data from an observational retrospective study including 1778 eligible patients suggest improved clinical outcomes in those receiving LBBAP compared with BVP [116]. Along this line, findings from a systematic review and meta-analysis including 3141 patients indicate reduced mortality and hospitalizations in LBBAP receivers compared to BVP [117]. Upgrading to LBBP is feasible and effective in CRT non-responders, achieving marked cardiac function improvement and better clinical outcomes, rendering it a reasonable alternative pacing strategy [102]. Table 5 summarizes clinical trials on CSP vs. CRT.

## 8. Conclusions

Due to lack of robust clinical evidence, the choice between CRT-D and CRT-P should be based on shared decision making and individualized risk assessment. The two step EP-guided approach could serve as a risk stratification tool. Factors that should be taken into account are age, etiology of heart failure, life expectancy, major comorbidities, poor renal function, and, of course, patient preference. Further studies are needed to identify subpopulations of patients with indications for CRT who will benefit the most from CRT-D implantation, justifying device-related risks compared to CRT-P alone.

## Figures and Tables

**Table 1:** Major CRT clinical trials.

Trial Name	Year	Population	Sample Size	Comparison	Endpoints
PATH-CHF [17]	1999	NYHA III-IV	42	Univentricular pacing vs. BiVP	Trends for improvement regarding *VO*2 max and 6MWT
MUSTIC [18]	2002	NYHA III LVEF < 35% QRS > 150 ms	67	BiVP vs. no pacing (sinus) BiVP vs. univentricular (patients with Af)	6MWT + 20% *VO*2 max + 10% LVEF + 5% Mitral regurgitation improved by 45–50%
MIRACLE [13]	2002	NYHA III-IV LVEF ≤ 35% QRS > 130 ms,	453	OMT vs. CRT	Improved quality of life, 6MWT, NYHA class, LVEF
MIRACLE-ICD [19]	2003	NYHA III-IV LVEF ≤ 35% QRS > 130 ms	369	BiVP + ICD vs. ICD	BiVP favorably affected quality of life, functional status, and exercise capacity No significant difference in LV function or survival
CONTAK-CD [20]	2003	NYHA) II -IV LVEF ≤ 35% QRS ≥ 120 ms	490	BiVP + ICD vs. ICD	6MWT + 20 m *VO*2 max + 0.8 mL/kg/min LVEF + 2.3%
COMPANION [9]	2004	NYHA III-IV LVEF ≤ 35% QRS ≥ 120 ms	1520	OMT vs. CRT/CRT-D	CRT-D reduced all-cause mortality by 36% CRT-P by 24%
CARE-HF [15]	2005	NYHA III-IV LVEF ≤35% QRS ≥ 120 ms	813	OMT vs. CRT	CRT-P reduced mortality and HF hospitalization
HOBIPACE [21]	2006	LVEF ≤ 40% Symptomatic bradycardia and impaired AV conduction	33	BiVP vs. Univentricular pacing	Favorable effects of BiVP on LV dimensions, LVEF, NT-proBNP levels, and functional status
MADIT-CRT [10]	2009	NYHA I-II LVEF ≤ 30% QRS ≥ 130 ms,	1820	CRT-D vs. ICD	Reduced HF events and improved LV function, especially in LBBB
REVERSE [14]	2008	NYHA I-II LVEF ≤ 40% QRS ≥ 120 ms	610	OMT vs. CRT	CRT-P improved LV function and reduced HF progression
RAFT [11]	2010	NYHA II-III LVEF ≤ 30% QRS ≥ 120 ms	1798	CRT-D vs. ICD	CRT-D reduced mortality and HF hospitalizations
RESET-CRT [22]	2023	NYHA II-IV LVEF ≤ 35% QRS ≥ 120 ms	3569	CRT-P vs. CRT-D	Non-inferior mortality with CRT-P vs. CRT-D

Abbreviations: NYHA, New York Heart Association; LVEF, left ventricular ejection fraction; HF, heart failure; MWT, minute walking distance; OMT, optimal medical treatment; CRT, cardiac resynchronization.

**Table 2:** Current recommendations regarding cardiac physiologic pacing.

**2021 ESC Guidelines on cardiac pacing and cardiac resynchronization therapy**		
**Recommendations**	Class ^a^	Level ^b^
**Sinus Rhythm**		
**SR and LBBB QRS morphology**		
CRT is recommended for symptomatic patients with HF in SR with LVEF ≤ 35%, QRS duration ≥ 150 ms, and LBBB QRS morphology despite OMT in order to improve symptoms and reduce morbidity and mortality.	I	A
CRT should be considered for symptomatic patients with HF in SR with LVEF ≤ 35%, QRS duration 130–149 ms, and LBBB QRS morphology despite OMT in order to improve symptoms and reduce morbidity and mortality.	IIa	B
**SR and Non-LBBB QRS morphology**		
CRT should be considered for symptomatic patients with HF in SR with LVEF ≤ 35%, QRS duration ≥ 150 ms, and non-LBBB QRS morphology despite OMT in order to improve symptoms and reduce morbidity.	IIa	B
CRT may be considered for symptomatic patients with HF in SR with LVEF ≤ 35%, QRS duration 130–149 ms, and non-LBBB QRS morphology despite OMT in order to improve symptoms and reduce morbidity and mortality.	IIb	B
QRS duration		
CRT is not indicated in patients with HF and QRS duration < 130 ms without an indication for RV pacing.	III	A
**Atrial Fibrillation**		
(1) In patients with HF with permanent AF who are candidates for CRT:		
(1A) CRT should be considered for patients with HF and LVEF < 35% in NYHA class III or IV despite OMT if they are in AF and have intrinsic QRS ≥ 130 ms, provided a strategy to ensure biventricular capture is in place, in order to improve symptoms and reduce morbidity and mortality.	IIa	C
(1B) AVJ ablation should be added in the case of incomplete biventricular pacing (<90–95%) due to conducted AF.	IIa	B
(2) In patients with symptomatic AF and an uncontrolled heart rate who are candidates for AVJ ablation (irrespective of QRS duration):		
(2A) CRT is recommended in patients with HFrEF.	I	B
(2B) CRT rather than standard RV pacing should be considered in patients with HFmrEF.	IIa	C
(2C) RV pacing should be considered in patients with HFpEF.	IIa	B
(2D) CRT may be considered in patients with HFpEF.	IIb	C
**Upgrade to CRT**		
Patients who have received a conventional pacemaker or an ICD and who subsequently develop symptomatic HF with LVEF < 35% despite OMT, and who have a significant proportion of DV pacing, should be considered for upgrade to CRT.	IIa	B
CRT rather than RV pacing is recommended for patients with HFrEF (<40%) regardless of NYHA class who have an indication for ventricular pacing and high-degree AVB in order to reduce morbidity. This includes patients with AF.	I	A
In patients who are candidates for an ICD and who have CRT indication, implantation of a CRT-D is recommended.	I	A
In patients who are candidates for CRT, implantation of a CRT-D should be considered after individual risk assessment and using shared decision-making.	IIa	B
LBBB = left bundle branch block; AF = atrial fibrillation; AVJ = atrioventricular junction; CRT = cardiac resynchronization therapy; EF = ejection fraction; HF = heart failure; HFrEF = heart failure with reduced ejection fraction (<40%); HFmrEF = heart failure with mildly reduced ejection fraction (40–49%); HFpEF = heart failure with preserved ejection fraction (≥50%) according to the 2021 ESC HF Guidelines; LVEF = left ventricular ejection fraction; NYHA = New York Heart Association; RV = right ventricular; ICD = implantable cardioverter-defibrillator; OMT = optimal medical therapy; AVB = atrioventricular block; NYHA = New York Heart Association. ^a^ Class of recommendation ^b^ Level of evidence		
**2022 AHA/ACC/HFSA Guideline for the Management of Heart Failure: A Report of the American College of Cardiology/American Heart Association Joint Committee on Clinical Practice** **Guidelines**		
**Recommendations**	**Class ^a^**	**Level ^b^**
**SR and LBBB QRS morphology**		
For patients who have LVEF ≤ 35%, sinus rhythm, left bundle branch block (LBBB) with a QRS duration ≥ 150 ms, and NYHA class II, III, or ambulatory IV symptoms on GDMT, CRT is indicated to reduce total mortality, reduce hospitalizations, and improve symptoms and QOL.	1	B-R
For patients who have LVEF ≤ 35%, sinus rhythm, LBBB with a QRS duration of 120 to 149 ms, and NYHA class II, III, or ambulatory IV symptoms on GDMT, CRT can be useful to reduce total mortality, reduce hospitalizations, and improve symptoms and QOL.	2a	B-NR
For patients who have LVEF ≤ 30%, ischemic cause of HF, sinus rhythm, LBBB with a QRS duration ≥ 150 ms, and NYHA class I symptoms on GDMT, CRT may be considered to reduce hospitalizations and improve symptoms and QOL.	2b	B-NR
**SR and non-LBBB QRS morphology**		
For patients who have LVEF ≤ 35%, sinus rhythm, a non-LBBB pattern with a QRS duration ≥150 ms, and NYHA class II, III, or ambulatory class IV symptoms on GDMT, CRT can be useful to reduce total mortality, reduce hospitalizations, and improve symptoms and QOL.	2a	B-R
For patients who have LVEF ≤ 35%, sinus rhythm, a non-LBBB pattern with QRS duration of 120 to 149 ms, and NYHA class III or ambulatory class IV on GDMT, CRT may be considered to reduce total mortality, reduce hospitalizations, and improve symptoms and QOL.	2b	B-NR
**High burden RVP**		
In patients with high-degree or complete heart block and LVEF of 36% to 50%, CRT is reasonable to reduce total mortality, reduce hospitalizations, and improve symptoms and QOL.	2a	B-R
For patients on GDMT who have LVEF ≤ 35% and are undergoing placement of a new or replacement device implantation with anticipated requirement for significant (>40%) ventricular pacing, CRT can be useful to reduce total mortality, reduce hospitalizations, and improve symptoms and QOL.	2a	B-NR
**Atrial fibrillation**		
In patients with AF and LVEF ≤ 35% on GDMT, CRT can be useful to reduce total mortality, improve symptoms and QOL, and increase LVEF if: (a) the patient requires ventricular pacing or otherwise meets CRT criteria and (b) atrioventricular nodal ablation or pharmacological rate control will allow for near 100% ventricular pacing with CRT.	2a	B-R
For patients with AF and LVEF ≤ 50%, if a rhythm control strategy fails or is not desired and ventricular rates remain rapid despite medical therapy, atrioventricular nodal ablation with implantation of a CRT device is reasonable.	2a	B-R
**No benefit**		
In patients with QRS duration < 120 ms, CRT is not recommended	No benefit	B-R
For patients with NYHA class I or II symptoms and non-LBBB pattern with QRS duration < 150 ms, CRT is not recommended.	No benefit	B-NR
For patients whose comorbidities or frailty limit survival with good functional capacity to <1 year, ICD and cardiac resynchronization therapy with defibrillation (CRT-D) are not indicated.	No benefit	C-LD

SR = sinus rhythm; LBBB = left bundle branch block; AF = atrial fibrillation; CRT = cardiac resynchronization therapy; LVEF = left ventricular ejection fraction; HF = heart failure; NYHA = New York Heart Association; RVP= right ventricular pacing; GDMT = guideline-directed medical therapy. ^a^ Class of recommendation. ^b^ Level of evidence.

**Table 3:** RCTs and observational data on the effect of CRT on mortality.

Study (Year)	Population	Study Period	Follow-Up	CRT-P (n)	CRT-D (n)	Outcomes
**RCTs**						
Køber (2016) [33]	NICM	2008–2014	67 months (median)	323	322	No difference in all-cause mortality between patients who received CRT and patients who did not.
Doran (2021) [56]	ICM and NICM	2000–2002	16.5 months (median)	617	595	The unadjusted and adjusted HRs for CRT-D versus CRT-P were both 0.84 (95% CI: 0.65–1.09) for all-cause mortality. NICM (n = 555): CRT-D reduced all-cause mortality compared to CRT-P (aHR: 0.54; 95% CI: 0.34–0.86) ICM (n = 657): CRT-D did not reduce all-cause mortality (aHR: 1.05; 95% CI: 0.77–1.44).
**Observational studies**						
Auricchio (2007) [17]	ICM and NICM	1994–2004	34 months (median)	572	726	CRT-D Non-significant decrease in mortality by 20% (HR 0.83, 95% CI 0.58–1.17, *p* = 0.284) Significant decrease in sudden cardiac death (HR 0.04, 95% CI 0.04–0.28, *p* < 0.002).
Morani (2013) [57]	ICM and NICM	2004–2007	55 months (median)	108	266	CRT-D significantly reduced all-cause mortality compared to CRT-P (73 CRT-D and 44 CRT-P patients died, rate 6.6 vs. 10.4%/year; log-rank test, *p* = 0.020).
Kutyifa (2014) [34]	ICM and NICM	2000–2011	28 months (median)	693	429	No mortality benefit in patients with CRT-D compared with CRT-P in the total cohort (HR 0.98, 95% CI 0.73–1.32, *p* = 0.884). ICM: CRT-D was associated with 30% risk reduction in all-cause mortality compared with CRT-P (HR 0.70, 95% CI 0.51–0.97, *p* = 0.03). NICM: No mortality benefit of CRT-D over CRT-P (HR 0.98, 95% CI 0.73–1.32, *p* = 0.894).
Looi (2014) [58]	ICM and NICM	2006–2010	29 months (mean)	354	146	CRT-D did not offer additional survival advantage over CRT-P.
Gold (2015) [59]	ICM and NICM	2004–2006	60 months (median)	74	345	10% mortality among CRT-D patients and 11.8% among CRT-P patients. CRT-ON was predicted to increase survival 22.8% (CRT-ON 52.5% vs. CRT-OFF 29.7%; HR 0.45; *p* = 0.21), leading to expected survival of 9.76 years (CRT-ON) versus 7.5 years (CRT-OFF).
Marijon (2015) [60]	ICM and NICM	2008–2010	222 months (mean)	535	1170	Increased mortality rate among CRT-P patients compared with CRT-D (relative risk 2.01, 95% CI 1.56–2.58). 95% of the excess mortality among CRT-P subjects was related to an increase in non-sudden death.
Reitan (2015) [61]	ICM and NICM	1999–2012	59 months (median)	448	257	Annual mortality differed between CRT-D and CRT-P (5.3% and 11.8%, respectively) After adjustment for covariates, CRT-D treatment (vs. CRT-P) was not associated with better long-term survival.
Munir (2016) [62]	ICM and NICM	2002–2013	40.8 months (median)	107	405	CRT-P patients had higher unadjusted mortality compared to CRT-D (HR = 1.54, 95% CI 1.15–2.08, *p* = 0.004). After adjustment (age at implant, sex, prior myocardial infarction, Charlson index, pre-implant LVEF, QRS morphology, drugs), this effect lost statistical significance (HR 1.18, 95% CI 0.78–1.77, *p* = 0.435).
Witt (2016) [63]	ICM and NICM	2000–2010	48 months (median)	489	428	CRT-D reduced all-cause mortality in ICM (aHR 0.74, 95% CI, 0.56–0.97; *p* = 0.03) but not in NICM (aHR 0.96, 95% CI, 0.60–1.51; *p* = 0.85).
Drozd (2016) [64]	ICM and NICM	2008–2012	36 months (mean)	544	251	No survival benefit in CRT-D patients compared with CRT-P (HR 1.09, 95% CI 0.84–1.41, *p* = 0.51).
Laish-Farkas (2017) [65]	Elderly with ICM and NICM	2006–2015	60 months (median)	142	104	In octogenarians, CRT-P is associated with similar morbidity and mortality outcomes to CRT-D.
Barra (2017) [35]	ICM and NICM	2002–2012	41.4 months (mean)	1270	4037	ICM: better survival with CRT-D vs. CRT-P (HR 0.76; 95% CI: 0.62–0.92, *p* = 0.005). NICM: no such difference was observed (HR: 0.92; 95% CI: 0.73–1.16, *p* = 0.49).
Martens (2017) [66]	ICM and NICM	2008–2015	38 months (mean)	361	326	All-cause mortality was higher in patients with CRT-P versus CRT-D (21% vs. 12%, *p* = 0.003), even after adjusting for baseline characteristics (HR 2.5; 95% CI 1.36–4.60, *p* = 0.003). Predominant non-cardiac mode of death in CRT-P recipients (n = 47 (71%) vs. n = 13 (38%) in CRT-D, *p* = 0.002).
Yokoshiki (2017) [67]	ICM and NICM	2011–2015	21 months (mean)	97	620	All-cause death or heart failure hospitalization diverged between the CRT-D and CRT-P groups with a rate of 22% vs. 42%, respectively, at 24 months (*p* = 0.0011). However, this apparent benefit of CRT-D over CRT-P was no longer significant after adjustment for covariates.
Leyva (2018) [68]	ICM and NICM	2000–2017	56.4 months (median)	999	551	CRT-D was associated with a lower total mortality (HR 0.72). ICM: CRT-D was associated with lower total mortality (HR 0.62), total mortality or HF hospitalization (HR 0.63), and total mortality or hospitalization for MACE (HR 0.59) (all *p* < 0.001). NICM: No differences in outcomes between CRT-D and CRT-P.
Döring (2018) [52]	Elderly with ICM and NICM	2008–2014	26 months (mean)	80	97	No significant difference in mortality between the two groups (*p* = 0.562).
Wang (2019) [51]	NICM	2002–2013	46 months (median)	42	93	CRT-P recipients had similar unadjusted mortality compared to CRT-D recipients (HR 1.04, 95% CI 0.56–1.93). Unchanged after adjusting for unbalanced covariates (HR 0.95, 95% CI 0.47–1.89) (LVEF, drugs, comorbidities).
Saba (2019) [69]	NICM	2007–2014	60 months	1236	4359	No difference between matched CRT-P and CRT-D recipients regarding the time to all-cause mortality (HR 0.90; 95% CI 0.74–1.09), any hospitalization (HR 1.13; 95% CI 0.98–1.30), and cardiac hospitalization (HR 0.98; 95% CI 0.83–1.17). CRT-P recipients had significantly lower medical costs at 12 and 24 months.
Barra (2019) [70]	ICM and NICM	2002–2013	30 months (mean)	534	1241	ICM: better survival with CRT-D vs. CRT-P (HR for mortality adjusted on propensity score and all mortality predictors: 0.76; 95% CI: 0.62 to 0.92; *p* = 0.005). NICM: no such difference was observed (HR: 0.92; 95% CI: 0.73 to 1.16; *p* = 0.49).
Leyva (2019) [50]		2009–2017	32.4 months (median)	24,811	25,273	Excess mortality was lower after CRT-D than after CRT-P in all patients (aHR 0.80, 95% CI 0.76–0.84]. in subgroups with (aHR 0.79, 95% CI 0.74–0.84) or without (aHR 0.82, 95% CI 0.74–0.91) ICM
Liang (2020) [30]	ICM and NICM	2005–2016	36 months (median)	126	219	No significant difference in the risk of mortality between CRT-D and CRT-P groups (HR 0.99, 95% CI 0.70–1.40, *p* = 0.95]. No significant difference between CRT-D and CRT-P in reducing mortality was observed in any pre-specified subgroups.
Gras (2020) [71]	ICM and NICM	2010–2017	913 ± 841 days	19,266	26,431	Higher all-cause mortality in CRT-P (11.6%) than CRT-D patients (6.8%) (HR 1.70, 95% CI 1.63–1.76, *p* < 0.001). No difference in mortality in NICM patients >75 years old with CRT-P and CRT-D (HR 0.93, 95% CI 0.80–1.09, *p* = 0.39). Higher mortality in NICM CRT-P patients <75 years old (HR 1.22, 95% CI 1.03–1.45, *p* = 0.02). Higher mortality with CRT-P than with CRT-D irrespectively of age in patients with ICM (<75 years old: HR 1.22, 95% CI 1.08–1.37, *p* = 0.01; ≥75 years old: HR 1.13, 95% CI 1.04–1.22, *p* = 0.003).
Huang (2020) [72]	ICM and NICM	2012–2013	27.7 months (mean)	237	362	SCD rate was 8.0% in CRT-P group and 3.3% in CRT-D group. No significant differences in all-cause death rate between the CRT-D and CRT-P groups (CRT-D vs. CRT-P, 20.4% vs. 19.4%, *p* = 0.840).
Schrage (2022) [73]	ICM and NICM	2000–2016	28.2 months (median)	880	1108	CRT-D was associated with lower 1- and 3-year all-cause mortality (HR:0.76, 95% CI:0.58–0.98; HR: 0.82, 95% CI: 0.68–0.99, respectively).
Hadwiger (2022) [22]	ICM and NICM	2014–2019	28.2 months (median)	847	2722	CRT-P treatment was not associated with inferior survival compared with CRT-D.
Farouq (2023) [74]	NICM	2005–2020	51.6 months (median)	2334	1693	CRT-D was associated with higher 5-year survival (HR 0.72 95% CI 0.61–0.85, *p* < 0.001). CRT-P was associated with higher mortality in age groups <60 years and 70–79 years.

Abbreviations: ICM, ischemic cardiomyopathy; NICM, non-ischemic cardiomyopathy; SCD, sudden cardiac death; HF, heart failure; MACE, major adverse cardiac events; aHR, adjusted hazard ratio; CI, confidence interval.

**Table 4:** Meta-analyses on the effect of CRT on mortality.

Meta-Analysis (year)	No. of Studies Included	Population (n)	CRT-P (n)	CRT-D (n)	Outcomes
**Ischemic and non-ischemic cardiomyopathy**					
Veres (2023) [31]	26 observational studies	128.030	55.469	72.561	CRT-D reduced all-cause mortality by 20% over CRT-P (aHR: 0.85; 95% CI: 0.76–0.94; *p* < 0.01] Not in NICM (HR: 0.95; 95% CI: 0.79–1.15; *p* = 0.19). Not in patients >75 years (HR: 1.08; 95% CI 0.96–1.21; *p* = 0.17).
**Non-ischemic cardiomyopathy**					
Patel (2021) [32]	7 observational studies	9.944	3.079	6.865	CRT-D was not significantly associated with a reduction in all-cause mortality in CRT-eligible patients with NICM (aHR 0.92, 95% CI, 0.83–1.03).
Al Sadawi (2023) [36]	13 observational and 2 RCTs	22.763	9.596	13.167	CRT-D was associated with lower all-cause mortality (log HR − 0.169, SE 0.055; *p* = 0.002) compared to CRT-P.
Liu (2023) [75]	9 observational and 2 RCTs	28.768	11.980	16.788	CRT-D was associated with a modest but statistically significant survival benefit compared to CRT-P (aHR 0.90 95% CI, 0.81–0.99).
Neto (2024) [76]	11 observational and 2 RCTs	61.326	7.338	9.108	CRT-D was associated with a significantly lower risk of all-cause mortality compared to CRT-P (pooled HR 0.74; 95% CI: 0.62–0.88; I^2^ = 84%). No statistically significant difference in mortality risk for patients > 75 years old (pooled HR 0.96; 95% CI: 0.811–1.15; I^2^ = 39%, *p* < 0.001).

Abbreviations: RCTs, randomized clinical trials; ICM, ischemic cardiomyopathy; NICM, non-ischemic cardiomyopathy; aHR, adjusted hazard ratio; CI, confidence interval.

**Table 5:** Clinical trials on CSP vs. CRT.

Trial Name	Year	Study Type	Population	Intervention	Comparator	Key Findings
His-SYNC [110]	2017	RCT	41	HBP	BiV CRT	Similar improvements in LV function; HBP had higher crossover (48%) due to technical limitations.
His-Alternative [111]	2021	RCT	50	HBP or LBBAP	BiV CRT	CSP showed non-inferior clinical response and better electrical resynchronization.
LBBP-RESYNC [109]	2022	RCT	40	LBBAP	BiV CRT	LBBAP showed greater LVEF improvement and QRS narrowing than BiV CRT.
HOT CRT [118]	2023	RCT	160	HBP	BiV CRT	HBP was superior to BiVP in LVEF; similar QRS narrowing and symptoms.
LEVEL-AT [99]	2023	Prospective, non-randomized	70	LBBAP	CRT cohort	LBBAP showed better LVEF recovery and symptom improvement compared to historical BiV CRT.
CONSYST CRT [119]	2025	RCT	134	CSP	BiV CRT	Non inferiority in clinical and echocardiographic response.

Abbreviations: RCT, randomized clinical trial; HBP, his bundle pacing; LBBAP, left bundle brunch area pacing; BiV, biventricular; CSP, conduction system pacing; CRT, cardiac resynchronization, LVEF, left ventricular ejection fraction; LV, left ventricular.

## Abbreviations

The following abbreviations are used in this manuscript:

CRT	Cardiac resynchronization therapy
RCTs	Randomized clinical trials
LVEF	Left ventricular ejection fraction
LBBB	Left bundle branch block
AF	Atrial fibrillation
RV	Right ventricular
OGMT	Optimal guideline-directed medical therapy
PVS	Programmed ventricular stimulation
NIRFs	Non-invasive risk factors
NIPS	Non-invasive programmed stimulation
EP	Electrophysiology
SCD	Sudden cardiac death
SMVT	Sustained monomorphic ventricular tachycardia
CSP	Conduction system pacing
HBP	His bundle pacing
LBBAP	Left bundle branch area pacing
CMR	Cardiac magnetic resonance
NICM	Non-ischemic cardiomyopathy
ICM	Ischemic cardiomyopathy
LGE	Late gadolinium enhancement
DCM	Dilated cardiomyopathy

## References

[1] Conrad N., Judge A., Tran J., Mohseni H., Hedgecott D., Crespillo A.P., Allison M., Hemingway H., Cleland J.G., McMurray J.J.V. (2018). Temporal trends and patterns in heart failure incidence: A population-based study of 4 million individuals. Lancet.

[2] Dunlay S.M., Weston S.A., Jacobsen S.J., Roger V.L. (2009). Risk Factors for Heart Failure: A Population-Based Case-Control Study. Am. J. Med..

[3] Virani S.S., Alonso A., Benjamin E.J., Bittencourt M.S., Callaway C.W., Carson A.P., Chamberlain A.M., Chang A.R., Cheng S., Delling F.N. (2020). Heart Disease and Stroke Statistics—2020 Update: A Report from the American Heart Association. Circulation.

[4] Leclercq C., Hare J.M. (2004). Ventricular resynchronization: Current state of the art. Circulation.

[5] Leclercq C., Kass D.A. (2002). Retiming the failing heart: Principles and current clinical status of cardiac resynchronization. J. Am. Coll. Cardiol..

[6] Lund L.H., Jurga J., Edner M., Benson L., Dahlström U., Linde C., Alehagen U. (2012). Prevalence, correlates, and prognostic significance of QRS prolongation in heart failure with reduced and preserved ejection fraction. Eur. Heart J..

[7] Shamim W., Francis D.P., Yousufuddin M., Varney S., Pieopli M.F., Anker S.D., Coats A.J. (1999). Intraventricular conduction delay: A prognostic marker in chronic heart failure. Int. J. Cardiol..

[8] Cleland J.G., Daubert J.C., Erdmann E., Freemantle N., Gras D., Kappenberger L., Tavazzi L. (2005). The effect of cardiac resynchro-nization on morbidity and mortality in heart failure. N. Engl. J. Med..

[9] Bristow M.R., Saxon L.A., Boehmer J., Krueger S., Kass D.A., De Marco T., Carson P., DiCarlo L., DeMets D., White B.G. (2004). Cardiac-Resynchronization Therapy with or without an Implantable Defibrillator in Advanced Chronic Heart Failure. N. Engl. J. Med..

[10] Moss A.J., Hall W.J., Cannom D.S., Klein H., Brown M.W., Daubert J.P., Estes N.A.M., Foster E., Greenberg H., Higgins S.L. (2009). Cardiac-Resynchronization Therapy for the Prevention of Heart-Failure Events. N. Engl. J. Med..

[11] Tang A.S., Wells G.A., Talajic M., Arnold M.O., Sheldon R., Connolly S., Hohnloser S.H., Nichol G., Birnie D.H., Sapp J.L. (2010). Cardiac-Resynchronization Therapy for Mild-to-Moderate Heart Failure. N. Engl. J. Med..

[12] Leyva F., Zegard A., Patel P., Stegemann B., Marshall H., Ludman P., de Bono J., Boriani G., Qiu T. (2023). Improved prognosis after cardiac resyn-chronization therapy over a decade. Europace.

[13] Abraham W.T., Fisher W.G., Smith A.L., Delurgio D.B., Leon A.R., Loh E., Kocovic D.Z., Packer M., Clavell A.L., Hayes D.L. (2002). Cardiac resynchronization in chronic heart failure. N. Engl. J. Med..

[14] Linde C., Abraham W.T., Gold M.R., John Sutton M., Ghio S., Daubert C., REVERSE Study Group (2008). Randomized trial of cardiac resynchronization in mildly symptomatic heart failure patients and in asymptomatic patients with left ventricular dysfunction and previous heart failure symptoms. J. Am. Coll. Cardiol..

[15] Cleland J., Daubert J., Erdmann E., Freemantle N., Gras D., Kappenberger L., Klein W., Tavazzi L. (2001). The CARE-HF study Steering Committee and Investigators The CARE-HF study (CArdiac REsynchronisation in Heart Failure study): Rationale, design and end-points. Eur. J. Heart Fail..

[16] Cleland J.G., Freemantle N., Erdmann E., Gras D., Kappenberger L., Tavazzi L., Daubert J. (2012). Long-term mortality with cardiac resynchronization therapy in the Cardiac Resynchronization-Heart Failure (CARE-HF) trial. Eur. J. Heart Fail..

[17] Auricchio A., Stellbrink C., Sack S., Block M., Vogt J., Bakker P., Mortensen P., Klein H., PATH-CHF Study Group (1999). The Pacing Therapies for Congestive Heart Fail-ure (PATH-CHF) study: Rationale, design, and endpoints of a prospective randomized multicenter study. Am. J. Cardiol..

[18] Cazeau S., Leclercq C., Lavergne T., Walker S., Varma C., Linde C., Garrigue S., Kappenberger L., Haywood G.A., Santini M. (2001). Effects of Multisite Biventricular Pacing in Patients with Heart Failure and Intraventricular Conduction Delay. N. Engl. J. Med..

[19] Young J.B., Abraham W.T., Smith A.L., Leon A.R., Lieberman R., Wilkoff B., Canby R.C., Schroeder J.S., Liem L.B., Hall S. (2003). Combined cardiac resynchronization and implantable cardioversion defibrillation in advanced chronic heart failure: The MIRACLE ICD Trial. JAMA.

[20] Higgins S.L., Hummel J.D., Niazi I.K., Giudici M.C., Worley S.J., Saxon L.A., Boehmer J.P., Higginbotham M.B., De Marco T., Foster E. (2003). Cardiac resynchronization therapy for the treatment of heart failure in patients with intraventricular conduction delay and malignant ventricular tachyarrhythmias. J. Am. Coll. Cardiol..

[21] Kindermann M., Hennen B., Jung J., Geisel J., Böhm M., Fröhlig G. (2006). Biventricular versus conventional right ventricular stimulation for patients with standard pacing indication and left ventricular dysfunction: The Homburg Biventricular Pacing Evaluation (HOBIPACE). J. Am. Coll. Cardiol..

[22] Hadwiger M., Dagres N., Haug J., Wolf M., Marschall U., Tijssen J., Katalinic A., Frielitz F.S., Hindricks G. (2022). Survival of patients undergoing cardiac resynchronization therapy with or without defibrillator: The RESET-CRT project. Eur. Heart J..

[23] Glikson M., Nielsen J.C., Kronborg M.B., Michowitz Y., Auricchio A., Barbash I.M., Barrabés J.A., Boriani G., Braunschweig F., Brignole M. (2021). 2021 ESC Guidelines on cardiac pacing and cardiac resynchronization therapy. Eur. Heart J..

[24] Merkely B., Hatala R., Wranicz J.K., Duray G., Földesi C., Som Z., Németh M., Goscinska-Bis K., Gellér L., Zima E. (2023). Upgrade of right ventricular pacing to cardiac resynchronization therapy in heart failure: A randomized trial. Eur. Heart J..

[25] Sideris S., Poulidakis E., Aggeli C., Gatzoulis K., Vlaseros I., Dilaveris P., Sotiropoulos I., Papoutis D., Xristakopoulos S., Manakos K. (2014). Upgrading pacemaker to cardiac resynchronization therapy: An option for patients with chronic right ventricular pacing and heart failure. Hell. J. Cardiol..

[26] Sridhar A.R.M., Yarlagadda V., Parasa S., Reddy Y.M., Patel D., Lakkireddy D., Wilkoff B.L., Dawn B. (2016). Cardiac Resynchronization Therapy: US Trends and Disparities in Utilization and Outcomes. Circ. Arrhythm. Electrophysiol..

[27] Yokoshiki H., Shimizu A., Mitsuhashi T., Furushima H., Sekiguchi Y., Manaka T., Nishii N., Ueyama T., Morita N., Nitta T. (2016). Trends and determinant factors in the use of cardiac resynchronization therapy devices in Japan: Analysis of the Japan cardiac device treatment registry database. J. Arrhythmia.

[28] Banks H., Torbica A., Valzania C., Varabyova Y., Rupel V.P., Taylor R.S., Hunger T., Walker S., Boriani G., Fattore G. (2017). Five year trends (2008–2012) in cardiac implantable electrical device utilization in five European nations: A case study in cross-country comparisons using administrative databases. Europace.

[29] Woods B., Hawkins N., Mealing S., Sutton A., Abraham W.T., Beshai J.F., Klein H., Sculpher M., Plummer C.J., Cowie M.R. (2015). Individual patient data network meta-analysis of mortality effects of implantable cardiac devices. Heart.

[30] Liang Y., Wang J., Yu Z., Zhang M., Pan L., Nie Y., Su Y., Ge J. (2020). Comparison between cardiac resynchronization therapy with and without defibrillator on long-term mortality: A propensity score matched analysis. J. Cardiol..

[31] Veres B., Fehérvári P., Engh M.A., Hegyi P., Gharehdaghi S., Zima E., Duray G., Merkely B., Kosztin A. (2023). Time-trend treatment effect of cardiac resynchronization therapy with or without defibrillator on mortality: A systematic review and meta-analysis. Europace.

[32] Patel D., Kumar A., Black-Maier E., Morgan R.L., Trulock K., Wilner B., Nemer D., Donnellan E., Tarakji K.G., Cantillon D.J. (2021). Cardiac Resynchronization Therapy With or Without Defibrillation in Patients with Nonischemic Cardiomyopathy: A Systematic Review and Meta-Analysis. Circ. Arrhythmia Electrophysiol..

[33] Køber L., Thune J.J., Nielsen J.C., Haarbo J., Videbæk L., Korup E., Jensen G., Hildebrandt P., Steffensen F.H., Bruun N.E. (2016). Defibrillator Implantation in Patients with Nonischemic Systolic Heart Failure. N. Engl. J. Med..

[34] Kutyifa V., Geller L., Bogyi P., Zima E., Aktas M.K., Ozcan E.E., Becker D., Nagy V.K., Kosztin A., Szilagyi S. (2014). Effect of cardiac resynchronization therapy with implantable cardioverter defibrillator versus cardiac resynchronization therapy with pacemaker on mortality in heart failure patients: Results of a high-volume, single-centre experience. Eur. J. Heart Fail..

[35] Barra S., Boveda S., Providência R., Sadoul N., Duehmke R., Reitan C., Borgquist R., Narayanan K., Hidden-Lucet F., Klug D. (2017). Adding Defibrillation Therapy to Cardi-ac Resynchronization on the Basis of the Myocardial Substrate. J. Am. Coll. Cardiol..

[36] Al-Sadawi M., Aslam F., Tao M., Salam S., Alsaiqali M., Singh A., Fan R., Rashba E.J. (2023). Is CRT-D superior to CRT-P in patients with nonischemic cardiomyopathy?. Int. J. Arrhythmia.

[37] Cleland J.G., Daubert J.-C., Erdmann E., Freemantle N., Gras D., Kappenberger L., Tavazzi L., The CARE-HF Study Investigators (2006). Longer-term effects of cardiac resynchronization therapy on mortality in heart failure [the CArdiac REsynchronization-Heart Failure (CARE-HF) trial extension phase]. Eur. Heart J..

[38] Gold M.R., Daubert J.C., Abraham W.T., Hassager C., Dinerman J.L., Hudnall J.H., Cerkvenik J., Linde C. (2013). Implantable defibrillators im-prove survival in patients with mildly symptomatic heart failure receiving cardiac resynchronization therapy: Analysis of the long-term follow-up of remodeling in systolic left ventricular dysfunction (REVERSE). Circ. Arrhythm. Electro-physiol..

[39] Gold M.R., Linde C., Abraham W.T., Gardiwal A., Daubert J.-C. (2011). The impact of cardiac resynchronization therapy on the incidence of ventricular arrhythmias in mild heart failure. Heart Rhythm..

[40] García-Lunar I., Castro-Urda V., Toquero-Ramos J., Mingo-Santos S., Moñivas-Palomero V., Mitroi C.D., Sánchez-García M., Pérez-Pereira E., Delgado H.E., Fernández-Lozano I. (2014). Ventricular Arrhythmias in Super-responders to Cardiac Resynchronization Therapy. Rev. Esp. Cardiol..

[41] Fang F., Yu C.-M. (2014). Shall CRT-D be downgraded to CRT-P in super-responders of cardiac resynchronization therapy?. Rev. Esp. Cardiol..

[42] Gatzoulis K.A., Tsiachris D., Arsenos P., Antoniou C.-K., Dilaveris P., Sideris S., Kanoupakis E., Simantirakis E., Korantzopoulos P., Goudevenos I. (2019). Arrhythmic risk stratification in post-myocardial infarction patients with preserved ejection fraction: The PRESERVE EF study. Eur. Heart J..

[43] Steffel J., Milosevic G., Hurlimann A., Krasniqi N., Namdar M., Ruschitzka F., Luscher T.F., Duru F., Holzmeister J., Hurlimann D. (2011). Characteristics and long-term outcome of echocardiographic super-responders to cardiac resynchronisation therapy: ‘real world’ experience from a single tertiary care centre. Heart.

[44] de Waard D., Manlucu J., Gillis A.M., Sapp J., Bernick J., Doucette S., Tang A., Wells G., Parkash R. (2019). Cardiac Resynchronization in Women: A Substudy of the Resynchronization-Defibrillation for Ambulatory Heart Failure Trial. JACC Clin. Electrophysiol..

[45] Zannad F., Ferreira J.P., Pocock S.J., Anker S.D., Butler J., Filippatos G., Brueckmann M., Ofstad A.P., Pfarr E., Jamal W. (2020). SGLT2 inhibitors in patients with heart failure with reduced ejection fraction: A meta-analysis of the EMPEROR-Reduced and DAPA-HF trials. Lancet.

[46] Mcmurray J.J.V., Packer M., Desai A.S., Gong J., Lefkowitz M.P., Rizkala A.R., Rouleau J.L., Shi V.C., Solomon S.D., Swedberg K. (2014). Angiotensin–Neprilysin Inhibition versus Enalapril in Heart Failure. PARADIGM-HF Investigators and Committees. N. Engl. J. Med..

[47] Pun P.H., Al-Khatib S.M., Han J.Y., Edwards R., Bardy G.H., Bigger J.T., Buxton A.E., Moss A.J., Lee K.L., Steinman R. (2014). Implantable Cardioverter-Defibrillators for Primary Prevention of Sudden Cardiac Death in CKD: A Meta-analysis of Patient-Level Data From 3 Randomized Trials. Am. J. Kidney Dis..

[48] Theuns D.A., Schaer B.A., Soliman O.I., Altmann D., Sticherling C., Geleijnse M.L., Osswald S., Jordaens L. (2010). The prognosis of implantable defibrillator patients treated with cardiac resynchronization therapy: Comorbidity burden as predictor of mortality. Europace.

[49] Goldenberg I., Vyas A.K., Hall W.J., Moss A.J., Wang H., He H., Zareba W., McNitt S., Andrews M.L., MADIT-II Investigators (2008). Risk Stratification for Primary Implantation of a Cardioverter-Defibrillator in Patients With Ischemic Left Ventricular Dysfunction. J. Am. Coll. Cardiol..

[50] Leyva F., Zegard A., Okafor O., de Bono J., McNulty D., Ahmed A., Marshall H., Ray D., Qiu T. (2019). Survival after cardiac resynchronization therapy: Results from 50 084 implantations. Europace.

[51] Wang Y., Sharbaugh M.S., Althouse A.D., Mulukutla S., Saba S. (2019). Cardiac resynchronization therapy pacemakers versus defibrillators in older non-ischemic cardiomyopathy patients. Indian Pacing Electrophysiol. J..

[52] Döring M., Ebert M., Dagres N., Müssigbrodt A., Bode K., Knopp H., Kühl M., Hindricks G., Richter S. (2018). Cardiac resynchronization therapy in the ageing population—With or without an implantable defibrillator?. Int. J. Cardiol..

[53] Acosta J., Fernández-Armenta J., Borràs R., Anguera I., Bisbal F., Martí-Almor J., Tolosana J.M., Penela D., Andreu D., Soto-Iglesias D. (2018). Scar Characterization to Predict Life-Threatening Arrhythmic Events and Sudden Cardiac Death in Patients with Cardiac Resynchronization Therapy: The GAUDI-CRT Study. JACC Cardiovasc. Imaging.

[54] Leyva F., Zegard A., Acquaye E., Gubran C., Taylor R., Foley P.W., Umar F., Patel K., Panting J., Marshall H. (2017). Outcomes of Cardiac Resynchronization Therapy with or Without Defibrillation in Patients with Nonischemic Cardiomyopathy. J. Am. Coll. Cardiol..

[55] Barra S., Providência R., Boveda S., Duehmke R., Narayanan K., Chow A.W., Piot O., Klug D., Defaye P., Gras D. (2018). Device complications with addition of defibrillation to cardiac resynchronisation therapy for primary prevention. Heart.

[56] Doran B., Mei C., Varosy P.D., Kao D.P., Saxon L.A., Feldman A.M., DeMets D., Bristow M.R. (2021). The Addition of a Defibrillator to Resynchronization Therapy Decreases Mortality in Patients With Nonischemic Cardiomyopathy. JACC Heart Fail..

[57] Morani G., Gasparini M., Zanon F., Casali E., Spotti A., Reggiani A., Bertaglia E., Solimene F., Molon G., Accogli M. (2013). Cardiac resynchronization therapy-defibrillator improves long-term survival compared with cardiac resynchronization therapy-pacemaker in patients with a class IA indication for cardiac resynchronization therapy: Data from the Contak Italian Registry. Europace.

[58] Looi K.-L., Gajendragadkar P.R., Khan F.Z., Elsik M., Begley D.A., Fynn S.P., Grace A.A., Heck P.M., Virdee M., Agarwal S. (2014). Cardiac resynchronisation therapy: Pacemaker versus internal cardioverter-defibrillator in patients with impaired left ventricular function. Heart.

[59] Gold M.R., Padhiar A., Mealing S., Sidhu M.K., Tsintzos S.I., Abraham W.T. (2015). Long-Term Extrapolation of Clinical Benefits Among Patients With Mild Heart Failure Receiving Cardiac Resynchronization Therapy: Analysis of the 5-Year Follow-Up From the REVERSE Study. JACC Heart Fail..

[60] Marijon E., Leclercq C., Narayanan K., Boveda S., Klug D., Lacaze-Gadonneix J., Defaye P., Jacob S., Piot O., Deharo J.-C. (2015). Causes-of-death analysis of patients with cardiac resynchronization therapy: An analysis of the CeRtiTuDe cohort study. Eur. Heart J..

[61] Reitan C., Chaudhry U., Bakos Z., Brandt J., Wang L., Platonov P.G., Borgquist R. (2015). Long-Term Results of Cardiac Resynchronization Therapy: A Comparison between CRT-Pacemakers versus Primary Prophylactic CRT-Defibrillators. Pacing Clin. Electrophysiol..

[62] Munir M.B., Althouse A.D., Rijal S., Shah M.B., Abu Daya H., Adelstein E., Saba S. (2016). Clinical Characteristics and Outcomes of Older Cardiac Resynchronization Therapy Recipients Using a Pacemaker versus a Defibrillator. J. Cardiovasc. Electrophysiol..

[63] Witt C.T., Kronborg M.B., Nohr E.A., Mortensen P.T., Gerdes C., Jensen H.K., Nielsen J.C. (2016). Adding the implantable cardioverter-defibrillator to cardiac resynchronization therapy is associated with improved long-term survival in ischaemic, but not in non-ischaemic cardiomyopathy. Europace.

[64] Drozd M., Gierula J., Lowry J.E., Paton M.F., Joy E., Jamil H.A., Cubbon R.M., Kearney M.T., Cairns D.A., Witte K.K. (2017). Cardiac resynchronization therapy outcomes in patients with chronic heart failure: Cardiac resynchronization therapy with pacemaker versus cardiac resynchronization therapy with defibrillator. J. Cardiovasc. Med..

[65] Laish-Farkash A., Bruoha S., Katz A., Goldenberg I., Suleiman M., Michowitz Y., Shlomo N., Einhorn-Cohen M., Khalameizer V. (2017). Morbidity and mortality with cardiac resynchronization therapy with pacing vs. with defibrillation in octogenarian patients in a real-world setting. Europace.

[66] Martens P., Verbrugge F.H., Nijst P., Dupont M., Nuyens D., Herendael H.V., Rivero-Ayerza M., Tang W.H., Mullens W. (2017). Incremental benefit of cardiac resynchronisation therapy with versus without a defibrillator. Heart.

[67] Yokoshiki H., Shimizu A., Mitsuhashi T., Furushima H., Sekiguchi Y., Manaka T., Nishii N., Ueyama T., Morita N., Okamura H. (2017). Survival and Heart Failure Hospitalization in Patients With Cardiac Resynchronization Therapy With or Without a Defibrillator for Primary Prevention in Japan—Analysis of the Japan Cardiac Device Treatment Registry Database. Circ. J..

[68] Leyva F., Zegard A., Umar F., Taylor R.J., Acquaye E., Gubran C., Chalil S., Patel K., Panting J., Marshall H. (2018). Long-term clinical outcomes of cardiac resynchronization therapy with or without defibrillation: Impact of the aetiology of cardiomyopathy. Europace.

[69] Saba S., McLaughlin T., He M., Althouse A., Mulukutla S., Hernandez I. (2019). Cardiac resynchronization therapy using pacemakers vs defibrillators in patients with nonischemic cardiomyopathy: The United States experience from 2007 to 2014. Heart Rhythm..

[70] Barra S., Duehmke R., Providência R., Narayanan K., Reitan C., Roubicek T., Polasek R., Chow A., Defaye P., Fauchier L. (2019). Very long-term survival and late sudden cardiac death in cardiac resynchronization therapy patients. Eur. Heart J..

[71] Gras M., Bisson A., Bodin A., Herbert J., Babuty D., Pierre B., Clementy N., Fauchier L. (2020). Mortality and cardiac resynchronization therapy with or without defibrillation in primary prevention. Europace.

[72] Huang W., Wu S., Vijayaraman P., Su L., Chen X., Cai B., Zou J., Lan R., Fu G., Mao G. (2020). Cardiac Resynchronization Therapy in Patients With Nonischemic Cardiomyopathy Using Left Bundle Branch Pacing. JACC Clin. Electrophysiol..

[73] Schrage B., Lund L.H., Melin M., Benson L., Uijl A., Dahlström U., Braunschweig F., Linde C., Savarese G. (2022). Cardiac resynchronization therapy with or without defibrillator in patients with heart failure. Europace.

[74] Farouq M., Rorsman C., Marinko S., Mörtsell D., Chaudhry U., Wang L., Platonov P.G., Borgquist R. (2023). Age-stratified comparison of prognosis in cardiac resynchronization therapy with or without prophylactic defibrillator for nonischemic cardiomyopathy-a nationwide cohort study. Europace.

[75] Liu F., Gao X., Luo J. (2023). An updated meta-analysis of cardiac resynchronization therapy with or without defibrillation in patients with nonischemic cardiomyopathy. Front. Cardiovasc. Med..

[76] Devesa Neto V., Costa G., Santos L.F., Costa A., Teixeira R., Goncalves L. (2024). Systematic review and meta-analysis comparing cardiac resynchronization therapy with versus without defibrillation in patients with non-ischemic cardiomyopathy. Europace.

[77] Gatzoulis K.A., Arsenos P., Trachanas K., Dilaveris P., Antoniou C., Tsiachris D., Sideris S., Kolettis T.M., Tousoulis D. (2018). Signal-averaged electrocardiography: Past, present. Signal-averaged electrocardiography: Past, present, and future. J. Arrhythm..

[78] Milaras N., Dourvas P., Doundoulakis I., Sotiriou Z., Nevras V., Xintarakou A., Laina A., Soulaidopoulos S., Zachos P., Kordalis A. (2023). Noninvasive electrocardiographic risk factors for sudden cardiac death in dilated ca rdiomyopathy: Is ambulatory electrocardiography still relevant?. Heart Fail. Rev..

[79] Stecker E.C., Vickers C., Waltz J., Socoteanu C., John B.T., Mariani R., McAnulty J.H., Gunson K., Jui J., Chugh S.S. (2006). Population-based analysis of sudden cardiac death with and without left ventricular systolic dysfunction: Two-year findings from the Oregon Sudden Unexpected Death Study. J. Am. Coll. Cardiol..

[80] Gorgels A.P., Gijsbers C., de Vreede-Swagemakers J., Lousberg A., Wellens H.J. (2003). Out-of-hospital cardiac arrest-the relevance of heart failure. The Maastricht Circulatory Arrest Registry. Eur. Heart J..

[81] Gatzoulis K.A., Dilaveris P., Arsenos P., Tsiachris D., Antoniou C.-K., Sideris S., Kolettis T., Kanoupakis E., Sideris A., Flevari P. (2021). Arrhythmic risk stratification in nonischemic dilated cardiomyopathy: The ReCONSIDER study design—A two-step, multifactorial, electrophysiology-inclusive approach. Hell. J. Cardiol..

[82] Laina A., Gatzoulis K., Soulaidopoulos S., Arsenos P., Doundoulakis I., Tsiachris D., Sideris S., Kordalis A., Tousoulis D., Tsioufis K. (2021). Time to reconsider risk stratification in dilated cardiomyopathy. Hell. J. Cardiol..

[83] Doundoulakis I., Tsiachris D., Kordalis A., Soulaidopoulos S., Arsenos P., Xintarakou A., Koliastasis L., Vlachakis P.K., Tsioufis K., Gatzoulis K.A. (2023). Management of Patients with Unexplained Syncope and Bundle Branch Block: Predictive Factors of Recurrent Syncope. Cureus.

[84] Demir E., Köse M.R., Şimşek E., Orman M.N., Zoghi M., Gürgün C., Nalbantgil S. (2025). Comparative analysis of implantable cardioverter-defibrillator efficacy in ischemic and non-ischemic cardiomyopathy in patients with heart failure. Sci. Rep..

[85] Frontera A., Prolic Kalinsek T., Hadjis A., Della Bella P. (2020). Noninvasive programmed stimulation in the setting of ven-tricular tachycardia catheter ablation. J. Cardiovasc. Electrophysiol..

[86] Kariki O., Antoniou C.-K., Mavrogeni S., Gatzoulis K.A. (2020). Updating the Risk Stratification for Sudden Cardiac Death in Cardiomyopathies: The Evolving Role of Cardiac Magnetic Resonance Imaging. An Approach for the Electrophysiologist. Diagnostics.

[87] Xintarakou A., Kariki O., Doundoulakis I., Arsenos P., Soulaidopoulos S., Laina A., Xydis P., Kordalis A., Nakas N., Theofilou A. (2022). The Role of Genetics in Risk Stratification Strategy of Dilated Cardiomyopathy. Rev. Cardiovasc. Med..

[88] Halliday B.P., Gulati A., Ali A., Guha K., Newsome S., Arzanauskaite M., Vassiliou V.S., Lota A., Izgi C., Tayal U. (2017). Association Between Midwall Late Gadolinium Enhancement and Sudden Cardiac Death in Patients With Dilated Cardiomyopathy and Mild and Moderate Left Ventricular Systolic Dysfunction. Circulation.

[89] Keil L., Chevalier C., Kirchhof P., Blankenberg S., Lund G., Müllerleile K., Magnussen C. (2021). CMR-Based Risk Stratification of Sudden Cardiac Death and Use of Implantable Cardioverter–Defibrillator in Non-Ischemic Cardiomyopathy. Int. J. Mol. Sci..

[90] Antonopoulos A.S., Xintarakou A., Protonotarios A., Lazaros G., Miliou A., Tsioufis K., Vlachopoulos C. (2024). Imagenetics for Precision Medicine in Dilated Cardiomyopathy. Circ. Genom. Precis. Med..

[91] Arbelo E., Protonotarios A., Gimeno J.R., Arbustini E., Barriales-Villa R., Basso C., Bezzina C.R., Biagini E., Blom N.A., de Boer R.A. (2023). 2023 ESC Guidelines for the management of cardiomyopathies. Eur. Heart J..

[92] Gigli M., Merlo M., Graw S.L., Barbati G., Rowland T.J., Slavov D.B., Stolfo D., Haywood M.E., Ferro M.D., Altinier A. (2019). Genetic Risk of Arrhythmic Phenotypes in Patients with Dilated Cardiomyopathy. J. Am. Coll. Cardiol..

[93] Gasperetti A., Carrick R.T., Costa S., Compagnucci P., Bosman L.P., Chivulescu M., Tichnell C., Murray B., Tandri H., Tadros R. (2022). Programmed Ventricular Stimulation as an Additional Primary Prevention Risk Stratification Tool in Arrhythmogenic Right Ventricular Cardiomyopathy: A Multinational Study. Circulation.

[94] Zeppenfeld K., Tfelt-Hansen J., de Riva M., Winkel B.G., Behr E.R., Blom N.A., Charron P., Corrado D., Dagres N., de Chillou C. (2022). 2022 ESC Guidelines for the management of patients with ventricular arrhythmias and the prevention of sudden cardiac death. Eur. Heart J..

[95] Trayanova N.A., Popescu D.M., Shade J.K. (2021). Machine Learning in Arrhythmia and Electrophysiology. Circ. Res..

[96] Ellenbogen K.A., Huizar J.F. (2012). Foreseeing super-response to cardiac resynchronization therapy: A perspective for clini-cians. J. Am. Coll. Cardiol..

[97] Daubert C., Behar N., Martins R.P., Mabo P., Leclercq C. (2017). Avoiding non-responders to cardiac resynchronization therapy: A practical guide. Eur. Heart J..

[98] Hummel J.D., Coppess M.A., Osborn J.S., Yee R., Fung J.W., Augostini R., Li S., Hine D., Singh J.P. (2016). Real-World Assessment of Acute Left Ventricular Lead Implant Success and Complication Rates: Results from the Attain Success Clinical Trial. Pacing Clin. Electrophysiol..

[99] Pujol-Lopez M., Jiménez-Arjona R., Garre P., Guasch E., Borràs R., Doltra A., Ferró E., García-Ribas C., Niebla M., Carro E. (2022). Conduction System Pacing vs Biventricular Pacing in Heart Failure and Wide QRS Patients. JACC Clin. Electrophysiol..

[100] Antoniou C.-K., Chrysohoou C., Manolakou P., Tsiachris D., Kordalis A., Tsioufis K., Gatzoulis K.A. (2025). Multipoint Left Ventricular Pacing as Alternative Approach in Cases of Biventricular Pacing Failure. J. Clin. Med..

[101] Dilaveris P., Antoniou C.-K., Chrysohoou C., Xydis P., Konstantinou K., Manolakou P., Kordalis A., Gatzoulis K., Tsioufis C. (2021). Comparative Trial of the Effects of Left Ventricular and Biventricular Pacing on Indices of Cardiac Function and Clinical Course of Patients with Heart Failure: Rationale and Design of the READAPT Randomized Trial. Angiology.

[102] Herweg B., Sharma P.S., Cano Ó., Ponnusamy S.S., Zanon F., Jastrzebski M., Zou J., Chelu M.G., Vernooy K., Whinnett Z.I. (2024). Arrhythmic Risk in Biventricular Pacing Compared with Left Bundle Branch Area Pacing: Results From the I-CLAS Study. Circulation.

[103] Fish J.M., Brugada J., Antzelevitch C. (2005). Potential Proarrhythmic Effects of Biventricular Pacing. J. Am. Coll. Cardiol..

[104] Nayak H.M., Verdino R.J., Russo A.M., Gerstenfeld E.P., Hsia H.H., Lin D., Dixit S., Cooper J.M., Callans D.J., Marchlinski F.E. (2008). Ventricular Tachycardia Storm After Initiation of Biventricular Pacing: Incidence, Clinical Characteristics, Management, and Outcome. J. Cardiovasc. Electrophysiol..

[105] Bradfield J.S., Shivkumar K. (2014). Cardiac Resynchronization Therapy–Induced Proarrhythmia. Circ. Arrhythmia Electrophysiol..

[106] Vouliotis A.I., Tsiachris D., Dilaveris P., Sideris S., Gatzoulis K.A. (2012). Cardiac Resynchronization Therapy and Proarrhythmia: Weathering the Storm. Hosp. Chron..

[107] Shukla G., Chaudhry G.M., Orlov M., Hoffmeister P., Haffajee C. (2005). Potential proarrhythmic effect of biventricular pacing: Fact or myth?. Heart Rhythm..

[108] Deif B., Ballantyne B., Almehmadi F., Mikhail M., McIntyre W.F., Manlucu J., Yee R., Sapp J.L., Roberts J.D., Healey J.S. (2018). Cardiac resynchronization is pro-arrhythmic in the absence of reverse ventricular remodelling: A systematic review and meta-analysis. Cardiovasc. Res..

[109] Wang Y., Zhu H., Hou X., Wang Z., Zou F., Qian Z., Wei Y., Wang X., Zhang L., Li X. (2022). Randomized Trial of Left Bundle Branch vs Biventricular Pacing for Cardiac Resynchronization Therapy. J. Am. Coll. Cardiol..

[110] Upadhyay G.A., Vijayaraman P., Nayak H.M., Verma N., Dandamudi G., Sharma P.S., Saleem M., Mandrola J., Genovese D., Tung R. (2019). His Corrective Pacing or Biventricular Pacing for Cardiac Resynchronization in Heart Failure. J. Am. Coll. Cardiol..

[111] Vinther M., Risum N., Svendsen J.H., Møgelvang R., Philbert B.T. (2021). A Randomized Trial of His Pacing Versus Biventricular Pacing in Symptomatic HF Patients with Left Bundle Branch Block (His-Alternative). JACC Clin. Electrophysiol..

[112] Archontakis S., Sideris K., Laina A., Arsenos P., Paraskevopoulou D., Tyrovola D., Gatzoulis K., Tousoulis D., Tsioufis K., Sideris S. (2021). His bundle pacing: A promising alternative strategy for anti-bradycardic pacing—Report of a single-center experience. Hell. J. Cardiol..

[113] Lustgarten D.L., Crespo E.M., Arkhipova-Jenkins I., Lobel R., Winget J., Koehler J., Liberman E., Sheldon T. (2015). His-bundle pacing versus biventricular pacing in cardiac resynchronization therapy patients: A crossover design comparison. Heart Rhythm..

[114] Vijayaraman P., Herweg B., Verma A., Sharma P.S., Batul S.A., Ponnusamy S.S., Schaller R.D., Cano O., Molina-Lerma M., Curila K. (2022). Rescue left bundle branch area pacing in coronary venous lead failure or nonresponse to biventricular pacing: Results from International LBBAP Collaborative Study Group. Heart Rhythm..

[115] Vijayaraman P., Ponnusamy S., Cano Ó., Sharma P.S., Naperkowski A., Subsposh F.A., Moskal P., Bednarek A., Dal Forno A.R., Young W. (2021). Left Bundle Branch Area Pacing for Cardiac Resynchronization Therapy: Results from the International LBBAP Collaborative Study Group. JACC Clin. Electrophysiol..

[116] Vijayaraman P., Sharma P.S., Cano Ó., Ponnusamy S.S., Herweg B., Zanon F., Jastrzebski M., Zou J., Chelu M.G., Vernooy K. (2023). Comparison of Left Bundle Branch Area Pacing and Biventricular Pacing in Candidates for Resynchronization Therapy. J. Am. Coll. Cardiol..

[117] Leventopoulos G., Travlos C.K., Anagnostopoulou V., Patrinos P., Papageorgiou A., Perperis A., Gale C.P., Gatzoulis K.A., Davlouros P. (2023). Clinical Out-comes of Left Bundle Branch Area Pacing Compared with Biventricular Pacing in Patients with Heart Failure Requiring Cardiac Resynchronization Therapy: Systematic Review and Meta-Analysis. Rev. Cardiovasc. Med..

[118] Vijayaraman P., Pokharel P., Subzposh F.A., Oren J.W., Storm R.H., Batul S.A., Beer D.A., Hughes G., Leri G., Manganiello M. (2023). His-Purkinje Conduction System Pacing Optimized Trial of Cardiac Resynchronization Therapy vs Biventricular Pacing: HOT-CRT Clinical Trial. JACC Clin. Electrophysiol..

[119] Pujol-López M., Graterol F.R., Borràs R., Garcia-Ribas C., Guichard J.B., Regany-Closa M., Jiménez-Arjona R., Niebla M., Poza M., Carro E. (2025). Clinical Response to Resynchronization Therapy: Conduction System Pacing vs. Biventricular Pacing: The CONSYST-CRT trial. JACC Clin. Electrophysiol..

